# Efficacy and Safety of a 3‐Weekly TS‐1 Adjuvant Regimen in Advanced Gastric Cancer: A Pilot Study

**DOI:** 10.1002/cam4.71079

**Published:** 2025-07-31

**Authors:** Jihong Bae, Joo‐Hwan Park, Young Saing Kim, Hee Kyung Ahn, Eun Kyung Cho, Dong Bok Shin, Ji‐Hyeon Park, Jun‐Young Yang, Woon Kee Lee, Sun Jin Sym

**Affiliations:** ^1^ Division of Medical Oncology, Department of Internal Medicine Gachon University Gil Medical Center Incheon Republic of Korea; ^2^ Department of Surgery Gachon University Gil Medical Center Incheon Republic of Korea

**Keywords:** 3‐weekly TS‐1, adjuvant therapy, advanced gastric cancer, compliance, recurrence‐free survival

## Abstract

**Background:**

TS‐1 at 80 mg/m^2^/day for 4 weeks followed by a 2‐week rest is standard adjuvant therapy for stage II/III Advanced Gastric Cancer (AGC). TS‐1 for 1 year (8 courses) is highly recommended. We investigated the efficacy and safety of an adjuvant 3‐weekly TS‐1 regimen for AGC.

**Methods:**

We analyzed 93 patients with stage II/III AGC who started 3‐weekly adjuvant TS‐1 therapy between Feb 2017 and May 2022 post‐gastrectomy with D2 lymphadenectomy. The 3‐weekly regimen was TS‐1 at 80 mg/m^2^/day for 2 weeks, followed by a 1‐week rest for 1 year (16 courses).

**Results:**

Among 93 patients, 12 (13%) had disease recurrence during follow‐up (median 24.6 months, range 4.2%–63.3%). Seven (9.5%) with stage II (*n* = 73) and five (25%) with stage III (*n* = 20) experienced recurrence. Kaplan–Meier analysis estimated that Recurrence‐Free Survival (RFS) rates at 1, 3, and 5 years were 92.0% (95% CI; 86.5%–97.9%), 84.7% (95% CI; 76.4%–93.9%), and 78.6% (95% CI; 65.8%–94.0%), respectively. Eighty patients (86%) completed the treatment; 25 (26.9%) needed dose reduction. Adverse events, mostly grade 1 or 2 diarrhea (28%) and nausea (20%), were manageable.

**Conclusion:**

Our study revealed that the 3‐weekly TS‐1 regimen as adjuvant therapy exhibited good efficacy and manageable toxicity. This regimen as an adjuvant therapy for AGC should be evaluated in future studies.

## 1 Introduction

Please follow the structure outlined below. Gastric cancer is the fifth most prevalent cancer worldwide according to the World Health Organization (WHO) [1]. Its incidence exhibits substantial geographic variation, with notably high rates in Eastern Asia (i.e., China, Korea, and Japan), Eastern Europe, and specific parts of South America [2]. Although surgical resection is the primary treatment for gastric cancer, studies have indicated that adjuvant chemotherapy can enhance survival outcomes in patients with locally advanced disease [3]. Among the various treatment regimens explored to prevent postoperative recurrence, such as the SWOG INT‐0116 trial (focusing on adjuvant chemoradiation in the United States), the MAGIC trial (which introduced perioperative chemotherapy, predominantly in the United Kingdom), and the FLOT4 trial (which established the FLOT regimen as the current standard for perioperative treatment in Western countries), TS‐1 monotherapy, in conjunction with the capecitabine and oxaliplatin combination regimen from the CLASSIC trial, has emerged as a standard therapy for stage II or III advanced gastric cancer (AGC), particularly in East Asia [4, 5, 6, 7, 8]. The ACTS‐GC trial reported superior survival rates with TS‐1 monotherapy compared to surgical resection alone [6]. Thus, there is a clear consensus on the necessity of adjuvant therapy in patients with stages II and III AGC. However, it is essential to acknowledge that the conventional TS‐1 regimen, involving 4 weeks of treatment followed by a 2‐week break, is associated with substantial side effects. Many patients encounter challenges in adhering to the prescribed medication regimen [9]. In the ACTS‐GC study, which evaluated adjuvant TS‐1 therapy in patients with resected AGC, specific dose‐limiting toxicities (DLTs) were identified. The most common grade 3 or higher toxicities included anorexia (6.0%), nausea (3.7%), and diarrhea (3.1%). These adverse events often led to dose reduction or treatment discontinuation, highlighting the tolerability concerns associated with the conventional 6‐weekly TS‐1 regimen [6]. In response to this concern, we introduced a modified 3‐weekly TS‐1 regimen, in which patients take a 2‐week medication cycle followed by a 1‐week break. Although numerous studies have investigated the efficacy and safety of this 3‐weekly TS‐1 administration, most have focused on patients with unresectable AGC, thus restricting the scope of their findings [10, 11]. Notably, a multicenter study conducted in the Republic of Korea involving a substantial patient cohort (*n* = 1372) reported significant findings regarding the efficacy of 3‐weekly TS‐1 monotherapy for patients with unresectable AGC [10]. The observed outcomes indicate that the modified 3‐weekly TS‐1 regimen, characterized by a relatively brief treatment period, may contribute to diminishing side effects and enhancing medication adherence with adjuvant therapy following curative resection. In the recently presented 5‐year follow‐up data of the JCOG1104 (OPAS‐1) trial, which compared adjuvant TS‐1 therapy regimens of 4 (6 months) and 8 courses (1 year) for patients diagnosed with p‐stage II AGC, a conclusion was reached that strongly recommended the use of an 8‐course regimen [12]. Building upon these foundations, our study postulated that a 3‐weekly TS‐1 regimen (duration: 1 year) as adjuvant therapy could yield efficacy and safety outcomes comparable to the conventional 6‐weekly TS‐1 regimen. Consequently, we analyzed the clinical outcomes of patients with stage II or III AGC who underwent adjuvant therapy following surgery. By assessing the modified TS‐1 regimen, we aimed to offer novel insights into adjuvant gastric cancer treatment with enhanced treatment compliance and minimal adverse events.

## 2 Material and Methods

### 2.1 Patients

Ninety‐three patients with stage II or III AGC were initiated on 3‐weekly adjuvant TS‐1 therapy after gastrectomy with D2 lymphadenectomy at the Gachon University Gil Medical Center, Incheon, Republic of Korea between February 2017 and May 2022. The eligibility criteria for this pilot study were as follows: at least 18 years of age; patients who had undergone radical gastrectomy with D2 lymphadenectomy; patients who were diagnosed with stage II or III disease according to the American Joint Committee on Cancer (AJCC)/Union for International Cancer Control (UICC) 8th edition of the gastric cancer staging system; an Eastern Cooperative Oncology Group (ECOG) performance status (PS) of 0–2, appropriate organ function, and no critical complications. This study was reviewed and approved by the Institutional Review Board of Gachon University Gil Medical Center (Incheon, Republic of Korea). Written informed consent was obtained from all patients.

### 2.2 Treatment Methods

TS‐1 was orally administered to all patients as adjuvant therapy. The 3‐weekly TS‐1 regimen consisted of 80 mg/m^2^/day (e.g., 120 mg/day for BSA ≥ 1.50 m^2^) of TS‐1 for 2 weeks, followed by a 1‐week break, with treatment continuing for either 1 year or 16 courses. The initial TS‐1 dose was determined based on body surface area (BSA), with patients receiving 120 mg/day (60 mg bid) for BSA ≥ 1.50 m^2^, 100 mg/day (50 mg bid) for BSA > 1.25 to < 1.50 m^2^, and 80 mg/day (40 mg bid) for BSA ≤ 1.25 m^2^.

Continuation of chemotherapy was determined based on hematologic parameters and adverse events (AEs). Treatment was maintained if absolute neutrophil count (ANC) was > 1000/μL and platelet count was > 75,000/μL. In cases where Grade 3 or higher AEs occurred, treatment was temporarily interrupted until recovery, after which it was resumed at a reduced dose level. Dose reductions followed a stepwise approach, where the TS‐1 dosage was adjusted from 120 to 100 mg/day, from 100 to 80 mg/day, and from 80 to 50 mg (25 mg bid)/day, as clinically indicated. Treatment discontinuation was considered in cases of persistent toxicity or based on the physician's assessment of organ function and overall patient condition.

### 2.3 Assessment of Efficacy and Toxicity

Adverse events were evaluated using the Common Terminology Criteria for Adverse Events (CTCAE), version 5.0. Recurrence‐Free Survival (RFS) was calculated from the date of initial treatment until disease progression or the last follow‐up visit.

### 2.4 Statistical Analysis

The R studio software tool (version 4.2.2) was used for all statistical analyses of the collected data and survival analysis using the Kaplan–Meier estimation. For survival analysis, right censoring was applied to account for patients who were lost to follow‐up or had not experienced the event of interest by the end of the study period. Specifically, patients who remained event‐free at their last follow‐up were right‐censored at their last recorded visit. In cases where patients were lost to follow‐up before experiencing the event, they were censored at the date of their last known clinical encounter. Kaplan–Meier estimates were used to calculate median survival times and survival probabilities at specific time points.

## 3 Results

### 3.1 Baseline characteristics of the patients

A total of 93 patients were included in this study, initiating adjuvant TS‐1 therapy between February 2, 2017 and May 23, 2022. The median age of the patients was 69 years (range: 31–87 years). Of these patients, 59 (63%) were male, while 34 (37%) were female. Subtotal gastrectomy was the predominant surgical approach performed in 74% of patients, as compared to those who underwent total gastrectomy. Primary tumors were predominantly located in the body and antrum or pylorus regions (*n* = 78, 84%). Conversely, the upper (cardia/fundus) and diffuse types were observed in nine (9.6%) and six patients (6.4%), respectively. Evaluation of HER2 status revealed positivity in only eight patients (8.6%). Regarding cancer staging, 73 patients (79%) were classified as stage II, whereas 20 (21%) were stage III, based on postsurgery pathology reports (Table 1).

**TABLE 1:** Baseline Characteristics of the Patients (*N* = 93).

Variable	N (%)
Age (IQR/Range)	69 (59, 78/31 ~ 87)
Gender	
F	34 (37%)
M	59 (63%)
ECOG	
0	71 (76%)
1	19 (20%)
2	3 (3.2%)
Resection	
Total	24 (26%)
Subtotal	69 (74%)
Location	
Cardia	7 (7.5%)
Fundus	2 (2.1%)
Body	31 (33%)
Antrum or pylorus	47 (51%)
Diffuse	6 (6.5%)
Differentiation	
Well	3 (3.2%)
Moderate	29 (31%)
Poorly	61 (65%)
Invasion	
Lymphovascular	80 (86%)
Perineural	54 (58%)
HER2 positive	8 (8.6%)
T stage	
T1	9 (9.7%)
T2	23 (25%)
T3	41 (44%)
T4a	17 (18%)
T4b	3 (3.2%)
N stage	
N0	31 (33%)
N1	25 (27%)
N2	32 (34%)
N3	5 (5.4%)
Stage (AJCC 8th edition)	
IIA	36 (39%)
IIB	37 (40%)
IIIA	14 (15%)
IIIB	6 (6%)

Abbreviations: ECOG, Eastern Cooperative Oncology Group; F, female; HER2, human epidermal growth factor receptor 2; IQR, interquartile range; M, male.

### 3.2 Clinical Outcomes of Patients

**FIGURE 1:**
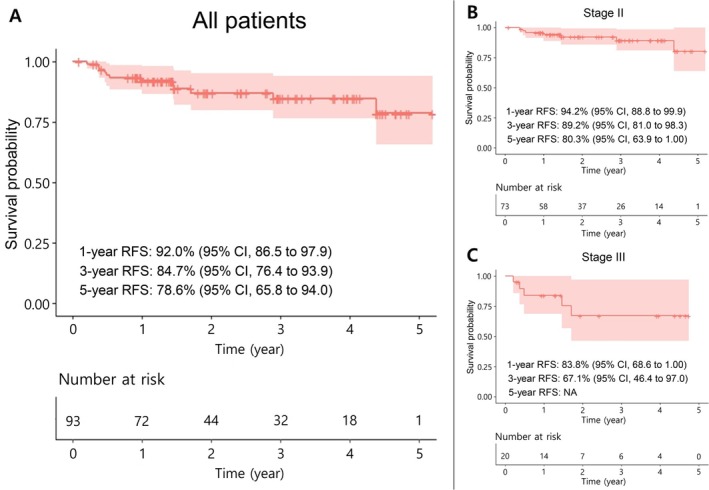
Kaplan–Meier estimates of Recurrence‐Free Survival for (A) all patients and (B) stage II or (C) III patients. RFS = Recurrence‐Free Survival; CI = Confidence Interval; NA = Not Available.

In our study, disease recurrence was observed in only 12 patients (13%) of the entire patient cohort during or after adjuvant therapy. The median duration of follow‐up was 24.6 months (range 4.2–63.3 months), and the data were analyzed retrospectively. RFS rates were assessed using the Kaplan–Meier estimation. The results indicated that a 3‐year RFS rate was 84.7% (95% CI; 76.4% to 93.9%), whereas a 5‐year RFS rate was 78.6% (95% CI; 65.8% to 94.0%) among all patients (Figure 1A). Subgroup analysis by cancer stage revealed that stage II patients had a 3‐year RFS rate of 89.2% (95% CI; 81.0% to 98.3%) and a 5‐year RFS rate of 80.3% (95% CI; 63.9% to 100%) (Figure 1B). Furthermore, for patients in stage III, the 3‐year RFS rate was 67.1% (95% CI; 46.4% to 97.0%) (Figure 1C); however, due to insufficient patient data for the 5‐year follow‐up period, it was not feasible to analyze the 5‐year RFS rate. In conclusion, our analysis revealed that modification of the adjuvant therapy schedule from a 6‐weekly (4 weeks on, 2 weeks off) to a 3‐weekly (2 weeks on, 1 week off) regimen led to improved survival rates as compared with previously published studies [6, 13]. Additionally, it is explicitly stated that this study is retrospective in nature, which may influence its limitations and interpretations. Further details, including a comparison of specific data, are addressed in the Discussion section.

### 3.3 Adverse Events and Compliance with Adjuvant TS‐1 Therapy

Data from 93 patients are presented to outline the adverse events (Table 2). Adverse events were graded according to the National Cancer Institute's Common Toxicity Criteria (version 5.0), with rare instances of grade 3 or 4 toxicities. Most cases were manageable and classified as grade 1 or 2 during the 3‐weekly TS‐1 administration period. Diarrhea and nausea were the most frequently observed adverse events, consistent with previous studies that showed high incidences of gastrointestinal symptoms [9, 13, 14, 15] (Table 2). Moreover, a small subset of four (4.3%) patients reported experiencing watery eyes, a well‐documented adverse event associated with lacrimal duct obstruction possibly induced by TS‐1 [16] (Table 2). None of these patients required intervention from ophthalmologists for watery eyes; consequently, all cases were evaluated as grade 1. Drug compliance and adherence were evaluated during follow‐up. Although dose reduction was implemented during adjuvant treatment in 25 patients (26.9%), 80 patients (86%) successfully completed adjuvant TS‐1 therapy after surgery (Table 3). The primary reasons for dose reduction were gastrointestinal symptoms such as diarrhea (28%), nausea (20%), and anorexia (16%) (Table 3). Thirteen patients were unable to complete the treatment, with four progressing to a palliative condition. Among the 12 patients who experienced progression, eight patients progressed after completing adjuvant therapy; six patients discontinued adjuvant treatment due to adverse events after confirming consent (Tables 3 and 4). Therefore, our findings confirmed that this 3‐weekly TS‐1 regimen induced low‐grade adverse events, increased compliance, and reduced the rate of dose reduction compared with previous studies [6, 13]. A detailed comparative analysis of the two regimens is provided in the Discussion section.

**TABLE 2:** Adverse events of patients with adjuvant S‐1 (*n* = 93).

Variable	Adverse events			
	Grade 1 (*n*)	Grade 2 (*n*)	Grade 3 (*n*)	Grade 4 (*n*)
Gastrointestinal				
Diarrhea	8 (8.6%)	6 (6.5%)	1 (1.1%)	—
Constipation	3 (3.2%)	2 (2.2%)	—	—
Abdominal pain	10 (10.8%)	1 (1.1%)	—	—
Nausea	11 (11.8%)	4 (4.3%)	—	—
Vomiting	4 (4.3%)	1 (1.1%)	—	—
Anorexia	10 (10.8%)	2 (2.2%)	—	—
Hyperbilirubinemia	5 (5.4%)	8 (8.6%)	1 (1.1%)	—
Hematology				
Anemia	—	5 (5.4%)	1 (1.1%)	—
Neutropenia	1 (1.1%)	—	—	—
Thrombocytopenia	—	1 (1.1%)	—	—
Neurology				
Headache	—	1 (1.1%)	—	—
Dermatology				
Pigmentation	4 (4.3%)	—	—	—
Pruritus	3 (3.2%)	1 (1.1%)	1 (1.1%)	—
Hand‐foot syndrome	—	—	—	—
General				
Weight loss	—	1 (1.1%)	—	—
Fatigue	1 (1.1%)	2 (2.2%)	1 (1.1%)	—
Stomatitis	4 (4.3%)	1 (1.1%)	—	—
Cheilitis	1 (1.1%)	—	—	—
Watering eyes	4 (4.3%)	—	—	—

**TABLE 3:** Compliance of S‐1 treatment (*n* = 93).

Variable	Patients
Completion of schedule (*n* = 93)	
Complete (full dose)	55 (59.1%)
Complete (dose reduction)	25 (26.9%)
Noncompletion	13 (14.0%)
Cause of noncompletion (*n* = 13)	
Progression	4 (30.8%)
Adverse events	6 (46.2%)
Others*	3 (23.1%)

* Two patients were diagnosed with subdural hematoma (SDH), a collection of blood between the dura mater and the arachnoid membrane, and underwent treatment, whereas one patient did not cooperate due to chronic alcoholism.

**TABLE 4:** Reasons for dose reduction (*n* = 25).

Variable	Patients
Gastrointestinal	
Anorexia	4 (16%)
Nausea	5 (20%)
Abdominal pain	3 (12%)
Constipation	1 (4.0%)
Diarrhea	7 (28%)
Hematology	
Anemia	1 (4.0%)
General	
Weight loss	1 (4.0%)
Fatigue	2 (8.0%)
Headache	1 (4.0%)

## 4 Discussion

TS‐1, developed by Taiho Pharmaceutical in Tokyo, Japan, combines tegafur, gimeracil, and oteracil in a specific ratio (1:0.4:1). Tegafur converts to fluorouracil in cells, gimeracil inhibits dihydropyrimidine dehydrogenase, and oteracil reduces gastrointestinal side effects [17, 18, 19, 20]. Clinical trials have shown a response rate greater than 40% in patients with advanced or recurrent gastric cancer [21, 22, 23]. To the best of our knowledge, the ACTS‐GC study represents the first extensive clinical trial of adjuvant chemotherapy in over 1000 patients who underwent D2 gastrectomy for gastric cancer in Japan [6, 13]. Since 2011, the National Health Insurance Corporation of Korea has approved adjuvant S‐1 treatment for stages II and III gastric cancer, based on evidence from the ACTS‐GC trial conducted in Japan [6, 13]. As the treatment has been approved for use in actual patients, studies evaluating the suitability of adjuvant TS‐1 therapy (standard 6‐weekly schedule) in curatively resected AGC patients have been conducted in Korea [15, 24]. Analysis of previous research findings has demonstrated that completing the full 1‐year course of adjuvant TS‐1 therapy without discontinuation, even in the presence of dose reduction or schedule modification due to adverse events, is crucial for achieving better clinical outcomes (longer survival rates or lower recurrence rates) [6, 9, 12, 15, 25]. In 2023, the results of the JCOG1104 (OPAS‐1) trial were published, which compared 4 courses (6 months) and 8 courses (1 year) of treatment in patients with stage II gastric cancer, supporting the importance of completing the 1‐year treatment for better clinical outcomes [12]. Taken together, the suitability of TS‐1 as an adjuvant therapy for gastric cancer has been substantiated through large‐scale research, confirming that 1‐year maintenance treatment is an appropriate treatment period.

The outcomes of our study validate the potential of a 3‐weekly TS‐1 regimen as an alternative to the standard 6‐weekly schedule for patients with stage II or III AGC requiring adjuvant therapy. Notably, this study revealed a tendency for improved RFS with 3‐weekly adjuvant TS‐1 therapy compared to the established ACTS‐GC data based on a 6‐weekly regimen, although the comparison may not be entirely fair due to differences in study design and patient population. Specifically, our study showed a 3‐year RFS rate of 84.7% (95% CI; 76.4%–93.9%), compared to the previous ACTS‐GC data of 72.2% (95% CI; 67.9% to 76.4%). Furthermore, the 5‐year RFS rate in our study was 78.6% (95% CI; 65.8% to 94.0%) compared to 65.4% (95% CI; 61.2% to 69.5%) in the ACTS‐GC data. In the context of stage II patients, our study demonstrated a 5‐year RFS rate of 80.3% (95% CI; 63.9% to 100%), aligning closely with the previously established result of 79.2% (95% CI; 73.8% to 84.6%) from the ACTS‐GC study. Collectively, our research provides compelling evidence in favor of the efficacy of a 3‐weekly TS‐1 schedule for a 1 year of maintenance.

In our study, the 3‐weekly adjuvant TS‐1 therapy exhibited notable advantages over the conventional 6‐weekly regimen. Specifically, it yielded a higher rate of 1‐year treatment completion and reduced the frequency of dose reduction owing to adverse events. We achieved an impressive 86% completion rate for the 1‐year schedule, a substantial improvement compared to the 65.8% completion rate observed in the prior large‐scale clinical trial, the ACTS‐GC trial, as well as the reported rates of 73% (71/97) in Japanese studies and 59% (29/49) in Korean studies [6, 9, 13, 15]. Although the study employed standard TS‐1 therapy (6‐weekly regimen), the 5‐year follow‐up results of the JCOG1104 (OPAS‐1) trial, unveiled in 2023, once again emphasized the higher recommendation for 8 courses (1 year) over 4 courses (6 months) [12]. For 4 courses of treatment, the 5‐year RFS rate was 85.6% and the 5‐year Overall Survival (OS) rate was 88.6%, compared with 87.7% and 89.7% for 8 courses, respectively [12]. The HR for cumulative recurrence incidence favored 8 courses at 1.38, with 38 recurrences for 4 courses and 28 for 8 courses [12]. Furthermore, our findings showed that the 3‐weekly TS‐1 regimen resulted in fewer instances of dose reduction during adjuvant therapy than the conventional 6‐weekly TS‐1 therapy. In the current study, of the 80 patients who completed the 1‐year treatment, only 25 (31.2%) required dose reduction. In contrast, in the ACTS‐GC trial, which applied 6‐weekly TS‐1 treatment, out of 340 patients who maintained the 1‐year treatment, 158 (46.5%) required dose reduction [13]. Similarly, in another Japanese study utilizing a 6‐weekly TS‐1 treatment, 57 of 97 patients (59%) experienced a dose reduction [9]. Consequently, our study, employing a 3‐weekly TS‐1 treatment, demonstrated a notably reduced rate of dose reduction attributable to patient‐presenting symptoms. To summarize, 3‐weekly adjuvant TS‐1 treatment is a suitable modification throughout the course of adjuvant therapy. Some studies have attempted to modify the dose or treatment duration of TS‐1, but the definitive possibility of these modifications has not been established [11, 12]. In contrast, the results of our study provide evidence that supports the feasibility of implementing schedule changes.

In addition, combination chemotherapy regimens such as SOX (ARTIST2) or S‐1/Docetaxel (JACCRO GC07) have demonstrated superior disease‐free survival (DFS) compared to TS‐1 monotherapy in certain patient populations (specifically, patients with stage III gastric cancer and those with lymph node‐positive disease) [26, 27, 28]. These important clinical trials have established significant reference standards in adjuvant therapy for gastric cancer, and their contributions should not be overlooked. However, these regimens are associated with increased toxicity and require careful patient selection based on clinical factors such as age, comorbidities, and tolerance levels. While acknowledging the valuable role of these combination therapies in the treatment landscape, our findings suggest that patients who may not tolerate combination regimens, particularly elderly patients with comorbidities and those with stage II or lower disease, could benefit from a modified 3‐weekly TS‐1 schedule as an effective yet less toxic alternative. This approach complements rather than replaces the established combination regimens, offering clinicians additional options within the spectrum of available treatments.

Nevertheless, when considering the 3 and 6‐weekly regimens, each has its own potential drawbacks. The 3‐weekly regimen may require more frequent clinical visits, potentially doubling the number of visits compared to the 6‐weekly schedule. This increased frequency of visits can be a significant inconvenience for patients, as it demands more time and effort for commuting and waiting at clinics. In contrast, the conventional 6‐weekly schedule involves fewer clinic visits but may result in a higher risk of dose reduction due to adverse events and lower treatment completion rates.

The present study had several limitations. First, this was a preliminary pilot study confined to a single center and involved a limited cohort (*n* = 93). Second, it is worth mentioning that the follow‐up period in this study was relatively short, and that the number of patients who completed a follow‐up period exceeding 5 years was quite limited. Therefore, ongoing patient follow‐up, as conducted in this study, is essential for data collection, with the expectation of deriving significant insights through subsequent analyses.

In conclusion, our findings suggest that a 3‐weekly TS‐1 regimen as adjuvant therapy holds potential as a valuable treatment option for patients with AGC. However, further research, including large‐scale studies and long‐term follow‐up, is warranted to confirm and refine these results. The evaluation of this regimen in future studies may provide additional insights into its role in AGC management and its potential to become a standard treatment approach.

## Author Contributions

Conceptualization: **Jihong Bae**, **Sun Jin Sym**.; data curation: **Jihong Bae**, **Joo‐Hwan Park**, **Young Saing Kim**, **Hee Kyung Ahn**, **Ji‐Hyeon Park**, **Jun‐Young Yang**, **Woon Kee Lee**.; formal analysis: **Jihong Bae**, **Joo‐Hwan Park**, **Young Saing Kim**, **Hee Kyung Ahn**, **Sun Jin Sym**.; supervision: **Eun Kyung Cho**, **Dong Bok Shin**, **Sun Jin Sym**.; writing – original draft: **Jihong Bae**, **Sun Jin Sym**.; writing – review and editing: **Jihong Bae**, **Sun Jin Sym**.

## Ethics Statement

The authors have nothing to report. This study was reviewed and approved by the Institutional Review Board of Gachon University Gil Medical Center (Incheon, Republic of Korea).

## Conflicts of Interest

The authors declare no conflicts of interest.

## Data Availability

The datasets generated and/or analyzed during the current study are available from the corresponding author on reasonable request. Due to privacy and ethical restrictions, individual patient‐level data are not publicly available. Any data that can be shared will be provided in de‐identified form, in accordance with institutional and ethical guidelines.
